# In Vitro and In Vivo Evaluation of Inhalable Ciprofloxacin Sustained Release Formulations

**DOI:** 10.3390/pharmaceutics15092287

**Published:** 2023-09-06

**Authors:** Changzhi Shi, Kewei Guo, Li Zhang, Yi Guo, Yu Feng, Sandra Cvijić, Dongmei Cun, Mingshi Yang

**Affiliations:** 1 Wuya College of Innovation, Shenyang Pharmaceutical University, Wenhua Road No. 103, Shenyang 110016, China; 2 Department of Pharmaceutical Technology and Cosmetology, Faculty of Pharmacy, University of Belgrade, Vojvode Stepe 450, 11221 Belgrade, Serbia; 3 Department of Pharmacy, Faculty of Health and Medical Sciences, University of Copenhagen, Universitetsparken 2, DK-2100 Copenhagen, Denmark

**Keywords:** respiratory infections, ciprofloxacin, sustained drug release, dry powder for inhalation, pulmonary drug exposure

## Abstract

Respiratory antibiotics delivery has been appreciated for its high local concentration at the infection sites. Certain formulation strategies are required to improve pulmonary drug exposure and to achieve effective antimicrobial activity, especially for highly permeable antibiotics. This study aimed to investigate lung exposure to various inhalable ciprofloxacin (CIP) formulations with different drug release rates in a rat model. Four formulations were prepared, i.e., CIP-loaded PLGA micro-particles (CHPM), CIP microcrystalline dry powder (CMDP), CIP nanocrystalline dry powder (CNDP), and CIP spray-dried powder (CHDP), which served as a reference. The physicochemical properties, drug dissolution rate, and aerosolization performance of these powders were characterized in vitro. Pharmacokinetic profiles were evaluated in rats. All formulations were suitable for inhalation (mass median aerodynamic diameter < 5 µm). CIP in CHPM and CHDP was amorphous, whereas the drug in CMDP and CNDP remained predominantly crystalline. CHDP exhibited the fastest drug release rate, while CMDP and CNDP exhibited much slower drug release. In addition, CMDP and CNDP exhibited significantly higher in vivo lung exposure to CIP compared with CHDP and CHPM. This study suggests that lung exposure to inhaled drugs with high permeability is governed by drug release rate, implying that lung exposure of inhaled antibiotics could be improved by a sustained-release formulation strategy.

## 1. Introduction

Respiratory infections are one of the major health threats that can cause high morbidity and mortality [1]. To combat respiratory infections, antibiotic treatment is a primary clinical approach [2]. Oral and/or parenteral (injectable) administration of antibiotics are two main administration routes to treat respiratory infections depending on the disease severity. Inhaled antibiotics have also been used to treat lung infections in cystic fibrosis patients [3]. Inhaled antibiotics can be delivered directly to the airways to the site of infection (targeted drug delivery), which may result in high local drug concentration, rapid onset of action, fewer side effects, and improved drug bioavailability [4]. However, it has often been observed that the concentration of an inhaled antibiotic in the epithelial lining fluid (ELF) declines too rapidly, especially for antibiotics with high permeability through the lung epithelium [5,6,7], such as fluoroquinolones. Pulmonary exposure to inhaled fluoroquinolones administered as an intratracheal bolus showed no discrepancy compared with systemic administration via the oral route or injection [8,9].

Most of the inhaled antibiotics available on the market today exhibit short-term therapeutic effects and require frequent drug administration. In respiratory infections, maintaining therapeutic concentrations of inhaled antibiotics in ELF, a susceptible site for pathogens, is a key factor in achieving the optimal therapeutic effect and suppressing antibiotic resistance [10]. One of the formulation strategies to maintain the desired concentration of inhaled antibiotics in the lungs may be the design of sustained-release inhaled formulations to prolong the duration of drug action, improve patients’ compliance, and reduce associated side effects [11,12]. Although sustained-release inhaled formulations have not yet been used in clinical practice, the research on utilizing this formulation strategy to prolong pulmonary drug exposure has been quite intensive due to the distinct advantages of such formulations [13,14,15].

In our previous study [9], ciprofloxacin (CIP), one of the potent fluoroquinolones, was used as a model compound, and CIP solutions were instilled intratracheally to rat lungs at three different rates (immediate, intermediate, and slow) to mimic different drug release rates of inhaled formulations. Subsequently, CIP concentrations in ELF and plasma as a function of time were determined, and the designed in silico ciprofloxacin-specific physiologically based pharmacokinetic model was used to identify the critical parameters influencing CIP systemic and local exposure following intratracheal administration at different instillation rates. This study suggested that slow delivery of CIP for an extended period of time may improve the bioavailability of CIP in ELF post-pulmonary administration. 

The aim of this study was to investigate lung exposure to four inhalable antibiotic dry powder formulations with different drug release rates following intratracheal administration to rats. We intended to determine whether the availability of the antibiotic in ELF post-pulmonary administration of dry powder formulations could be increased by extending the drug release rate. In this study, we used CIP as a model antibiotic. CIP was selected based on its two forms, i.e., water-soluble ciprofloxacin hydrochloride (CIPH), and poorly water-soluble ciprofloxacin free base (CIPB). To investigate lung exposure to dry powder formulations with different dissolution rates, two kinds of formulation strategies were used. One was to load water-soluble CIPH in sustained-release microparticles, and the other was to micronize poorly water-soluble CIPB crystals into micron sizes, i.e., microcrystal-based dry powder (CMDP). CIPB was also further micronized to less than 1 µm, i.e., nanocrystal-based dry powder (CNDP) with an expectation that the dissolution rate of CNDP was slower than CMDP but higher than the spray-dried CIPH powder (CHDP). Following physicochemical characterization, assessment of aerosol performance, and an in vitro drug release test, the pharmacokinetic profiles of the four dry powder formulations were determined following intratracheal insufflation to rats.

## 2. Materials and Methods

### 2.1. Materials

PLGA (Resomer^®^ 502H) was purchased from Evonik industries (Darmstadt, Germany). CIPH was obtained from Meilun Biotechnology (Dalian, Liaoning, China). Breezhaler^®^ (Novartis, Basel, Switzerland) was procured from the local pharmacy. Urea assay kits were purchased from Jiancheng Bioengineering Institute (Nanjing, Jiangsu, China). Acetonitrile and methanol were provided by Concord Technology (Tianjin, China). Deionized water was supplied by Milli-Q (Millipore^®^, Billerica, MA, USA). Other chemicals were of analytical grade. 

### 2.2. Formulation Preparation 

CHDP and CHPM were prepared by the spray-drying method [16]. Briefly, a B-290 spray dryer (Büchi, Flawil,Switzerland) with a 2-fluid nozzle (1.5 mm) and a 3-fluid nozzle (0.7/2.0/2.8 mm) in closed mode (B-295, B-296) was used for this purpose. For CHPM, 0.5 g PLGA was dissolved in 20 mL dichloromethane (Solution A) and 100 mg CIPH was dissolved in 5 mL water (Solution B), and the two solutions were passed through a 3-fluid nozzle (Inner Solution B: Outer Solution A, 1:4 *v*/*v*, 4 mL/min). The following conditions were applied: inlet temperature 60 °C (outlet temperature 43 ± 2 °C), aspiration rate 40 m^3^/h, atomization airflow 742 L/h. CHDP was prepared by spray drying of CIPH (1.05% *w*/*v*) in ethanol solution (30% *v*/*v*, 4 mL/min), at an inlet temperature of 130 °C (outlet temperature 78 ± 3 °C), aspiration rate 40 m^3^/h, and atomization airflow 414 L/h. 

CMDP and CNDP were prepared using the anti-solvent precipitation ultra-sonication method [17,18]. In the first step, CIPH solution (0.1 M, 1 mL) was added to sodium hydroxide solution (0.02 M, 5 mL) with a pipette under ultrasonic irradiation Biosafer 650-92 (Saifei Co., Ltd., Shanghai, China). The resultant precipitate was washed and resuspended with deionized water (10 mg/mL, CIPB microcrystal suspension, MCS). A similar method was used to prepare CIPB nanocrystal suspension (NCS), but an anti-solvent (isopropanol, 15 mL) was added to further inhibit the nucleation process. MCS was then freeze-dried for 24 h in a freeze-drier (Xinzhi Biotechnology Co., Ltd., Ningbo, Zhejiang, China) and NCS was also solidified by spray drying the same as CHDP. Products were stored in a desiccator until further analysis. 

### 2.3. Powder Morphology

The particle morphology of all prepared formulations was studied by scanning electron microscopy (SEM) (S4700, Hitachi, Japan). Prior to imaging, samples were dispersed, dried, and coated with gold at 10 mA for 30 s using a sputtering device. The samples were then examined, and the images were captured using the scanning electron microscope at an accelerating voltage of 5.0 kV.

### 2.4. Powder X-ray Diffraction 

The Powder X-ray Diffraction (PXRD) patterns of the supplied raw material and the prepared formulations were investigated using an X-ray diffractometer (Haoyuan Instrument, Dandong, Liaoning, China). All the samples were analyzed at a scan angular speed of 5°/min over the range (2 θ, 5°–40°) with a slit-detector radiation source of Cu-Kα (40 KV, 30 Ma). The measurements were performed in triplicate.

### 2.5. Aerosol Performance 

A Next-Generation Impactor (NGI, HRH-ZJQ-160, Hui Rong He Technology, Beijing, China) equipped with a USP throat was used to assess the in vitro aerosolization performance of the prepared dry powders. As previously described [19], dry powder (2.5 mg, equivalent ciprofloxacin) was filled into a hard capsule (hydroxypropyl methylcellulose, size 3, Capsugel, Suzhou, Jiangsu, China) and dosed using the Breezhaler^®^ device (Novartis, Basel, Switzerland). Prior to the test, the inhaler was shaken and the capsule was pierced, and then the activated capsule was aerosolized by the airstream through the impactor (P3/P2 < 0.5, 4 kPa, 60 L/min, 4 s). Particles deposited on different stages of the NGI (S1–S7), micro-orifice collector (MOC), and mouthpiece were collected by rinsing with the HPLC mobile phase and then transferred to volumetric flasks. The amount of the drug collected in each part of the NGI assembly was quantified using the HPLC method [9]. Each sample was quantified in triplicate. In vitro aerodynamic performance of dry powders was expressed in terms of the emitted fraction (EF), mass median aerodynamic diameter (MMAD), and fine particle fraction (FPF). The EF is the percentage of the loaded drug dose released from the capsule and aerosolized from the inhaler to deposit in the NGI. The FPF represents the ratio (% *w*/*w*) between the amount of drug particles with an aerodynamic diameter below 5 µm and the amount of the drug released from the inhaler. MMAD refers to the median particle diameter of the particles deposited in the NGI (as the cut-off diameter at 50% *w*/*w* of the deposited particles).

### 2.6. Drug Release Study 

To evaluate the in vitro drug release profiles, the prepared dry powders were accurately weighed, added in glass vials (10 mL deionized water, pH 7.0), and incubated (37 °C, 80 rpm) in a thermostatic oscillator (DSHZ-300A, Peiying Experimental Apparatus Co., Ltd., Taicang, Jiangsu, China) [20]. At predetermined time points (0.25, 0.5, 1, 2, 4, 6, 8, 10 h), a 1 mL sample of the medium was withdrawn and replaced with fresh pre-heated medium. After filtration, the drug content was determined by high-performance liquid chromatography (HPLC). All samples were tested in triplicate. Results are expressed as mean data and standard deviation (SD) for the cumulative percent of the drug released over time. 

### 2.7. Pharmacokinetic Study in Rats 

Sprague Dawley rats (male, 200–220 g) were commercially obtained from Changsheng Biotechnology Company (Benxi, Liaoning, China). The experimental protocol was approved by the local Animal Ethics Committee of Shenyang Pharmaceutical University (No. SYPU.IACUC.C2020.063030). 

The study was performed according to the previously described procedure [8]. Briefly, the tested formulations were insufflated into rats’ airways using a Model DP-4 aerosolizer (Penn-Century Inc., Wyndmoor, PA, USA). Before and after dosing, the device was weighed to obtain a total drug dose of 20 mg/kg (CHDP, CMDP, CNDP), or 2 mg/kg (CHPM). At predetermined time points (0.25, 0.5, 1, 2, 4, and 6 h), BALF (bronchoalveolar lavage fluid) and blood samples were immediately collected and centrifuged (13,800× *g*, 5 min). The initial drug distribution in the rats’ lungs was also determined by surgical collection from the lungs, according to the previously described method [19].

The protein precipitation method was used to determine the CIP content in the collected samples [21]. In brief, levofloxacin (0.5 μg/mL, internal standard) was added to 200 μL of the sample (plasma, BALF) and vortex for 1 min. Then, methanol and perchloric acid (5% *w*/*v*) were added, vortexed for 1 min, and centrifuged (13,800× *g*, 10 min) to collect the supernatant as HPLC samples. The CIP concentration in the ELF was estimated from the CIP concentration in the BALF corrected by a dilution factor representing the ratio between urea content in plasma and in the BALF [8]. Pharmacokinetic parameters were calculated using PKPlus^™^ software (version 9.8.1: Simulations Plus Inc., Lancaster, CA, USA), and they were expressed as AUC of ELF vs. AUC of plasma (AUC_EPR_, Equation (1)), apparent pulmonary bioavailability (AppELF, Equation (2)), and the pulmonary target index (PTI, Equation (3)) [22].

(1) $${\mathrm{AUC}}_{\mathrm{EPR}}=\frac{{\left({AUC}_{ELF}\right)}_{i.t.}\times {Dose}_{i.v.}}{{\left({AUC}_{plasma}\right)}_{i.v.}\times {Dose}_{i.t.}}$$

(2) $$\mathrm{AppELF}=\frac{{\left({AUC}_{ELF}\right)}_{i.t.}\times {Dose}_{i.v.}}{{\left({AUC}_{ELF}\right)}_{i.v.}\times {Dose}_{i.t.}}$$

(3) $$\mathrm{PTI}=\frac{{\left({AUC}_{ELF}/{AUC}_{plasma}\right)}_{i.t.}}{{\left({AUC}_{ELF}/{AUC}_{plasma}\right)}_{i.v.}}$$

### 2.8. HPLC Analysis

In this study, samples were analyzed by a Hitachi Chromatographic System (5410 UV-detector, 297 nm, Tokyo, Japan), using a C18 column (5 μm, 250 mm, 4.6 mm ID, Thermo-Fisher Scientific, San Jose, CA, USA). The mobile phase (1.00 mL/min) consisted of methanol, acetonitrile, 0.01 M phosphoric acid, and 0.5 M tetrabutylammonium bromide (30:30:430:10, *v*/*v*/*v*/*v*). The drug concentration was calculated using a calibration curve whose linearity (R^2^ > 0.999) was confirmed in a concentration range of 0.2–160 µg/mL. The inter- and intra-day precision and accuracy were less than 5% (10, 50, 150 µg/mL), and the limit of detection (LOD) and limit of quantitation (LOQ) were 40 ng/mL and 200 ng/mL, respectively.

### 2.9. Statistical Analysis

The results are indicated with the appropriate number of replicates (n) and represented as the mean value ± standard. Statistics were carried out using GraphPad Prism (version 8.0.2). *p*-values below 5% (*p* < 0.05) were considered statistically significant, as determined by analysis of variance (ANOVA) followed by a *t*-test.

## 3. Results and Discussion

### 3.1. Physicochemical Properties

**Figure 1:**
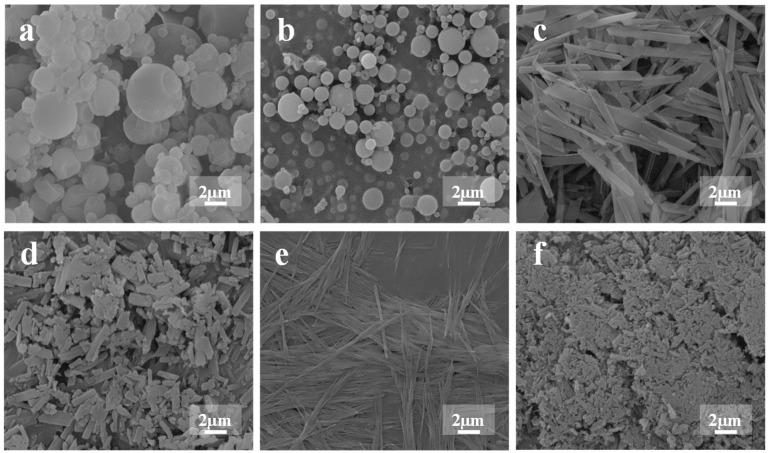
SEM images of CHDP (**a**), CHPM (**b**), MCS (**c**), CMDP (**d**), NCS (**e**), CNDP (**f**).

SEM images of the tested formulations (Figure 1) revealed that CHDP and CHPM consist of spherical particles. The particle sizes in CHDP are somewhat larger than in CHPM, but in both formulations, the particle sizes are in the range of 1–5 µm. The particles in MCS and NCS are needle shaped, with the particles in MCS being thicker than in NCS. This may explain why the primary particle sizes in CMDP are larger than those in CNDP. Both CMDP and CNDP are aggregates consisting of short rod-shaped particles, although the particles in CNDP are difficult to detect under the applied SEM resolution. The differences in the morphology of CMDP and CNDP particles can be attributed to different drying principles of freeze drying and spray drying, respectively.

**Figure 2:**
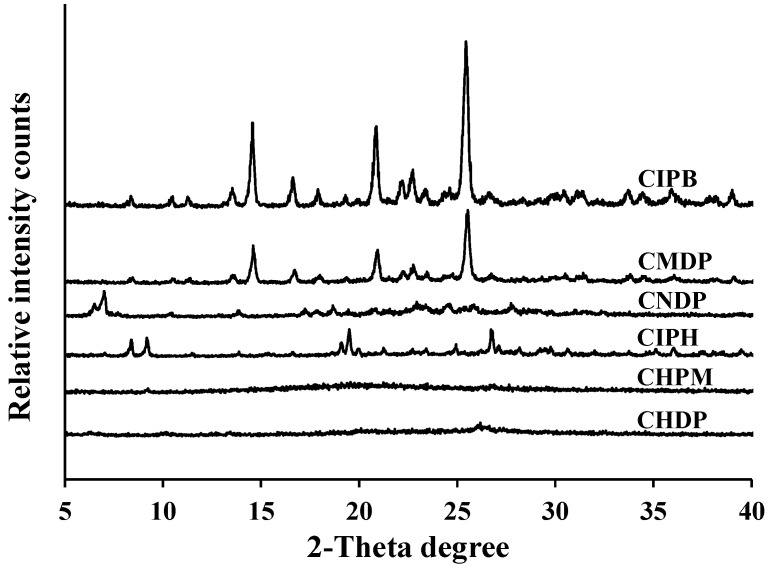
Representative PXRD patterns of CIPB, CIPH, CMDP, CNDP, CHPM, and CHDP.

XRPD patterns (Figure 2) revealed that CHPM and CHDP contain partly amorphous and partly crystalline drugs, with a higher proportion of crystalline residual drugs in CHDP than in CHPM. However, the percentage of the residual crystals in these two formulations is difficult to identify because the amorphous drug has been highly mixed with a crystalline drug and their peaks overlap each other. However, this result suggests that CIPH is highly dispersed in the PLGA matrix of CHPM post-spray drying, which is in good agreement with other studies [23]. Similarly, other researchers also reported that the spray drying process could result in the presence of amorphous CIPH in the dry powders [24,25,26], which is in close agreement with our amorphous results.

In contrast, the drug in CMDP and CNDP is mainly present in a crystalline form, although the crystallinity of CNDP samples seems to be much weaker than CMDP. This last observation can be attributed to the presence of crystals in a nano-size range. The PXRD pattern of CMDP is similar to that of CIPB, suggesting that the crystalline drug form remained unchanged after processing from MCS to CMDP. However, the PXRP pattern of CNDP is different from CIPB, implying that the crystalline form of CIPB was altered upon processing from NCS to CNDP. 

### 3.2. Aerosol Performance 

**Figure 3:**
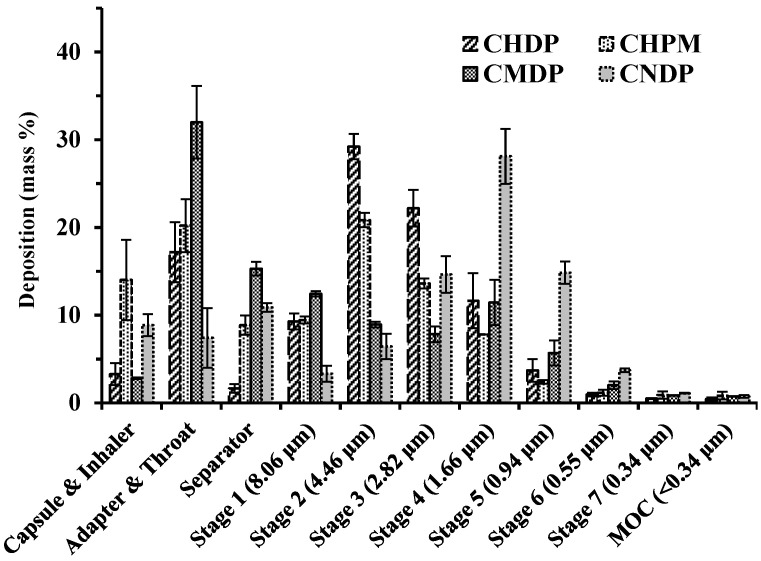
Distribution of particle deposition across components of the NGI assembly for CHDP, CHPM, CMDP, and CNDP. Mean ± SD, n = 3.

Drug delivery from dry powders for inhalation (DPIs) requires some inspiratory force to efficiently aerosolize powder from the inhaler, yet this can be difficult for patients suffering from lung diseases. Therefore, one of the challenges in the development of DPIs is to produce dry powder formulations with adequate mechanical properties to withstand handling, but at the same time are loose enough to allow efficient aerosolization during inhalation [27,28]. Moreover, it is important to achieve adequate DPI aerosolization performance without the need for high lung capacity and inspiratory flow. For this reason, a mid-region airflow of 60 L/min was selected for the NGI assay to test the aerosolization performance of the four dry powder formulations. The results are shown in Figure 3 and Table 1. 

Figure 3 illustrates that CMDP particles largely deposit in the adapter and throat, with an observable decline in deposition in later NGI stages. CHPM shows bimodal deposition, with a large number of particles deposited in the adapter and throat, and in Stage 2 of the NGI. A bimodal deposition pattern is also evident for CHDP and CNDP, but with higher fraction of deposited particles in the later NGI stages than in the pre-stage components of the impactor assembly. 

The data in Table 1 show that the EF values for all formulations are rather high regardless of the amount of DPIs filled in capsules. The MMAD values for the four formulations are approximately 2~4 µm, indicating that all tested DPIs are suitable for pulmonary drug delivery. The MMADs for CHDP and CHPM are slightly higher than for CMDP, while CNDP shows the smallest MMAD among the tested formulations (but the bulk densities are different, CHDP 153.90 mg/mL, CHPM 166.70 mg/mL, CMDP 31.23 mg/mL, CNDP 200.00 mg/mL). These results may explain the highest FPF for CNDP among the four formulations. Another observation is that FPF for CHDP is larger than for CHPM and CMDP, although their MMAD values are similar. This may be explained by a relatively large fraction of CMDP and CHPM particles deposited in the adapter and throat compartment. As a result, CMDP exhibited the lowest FPF among the four formulations.

### 3.3. In Vitro Drug Release Profiles

**Figure 4:**
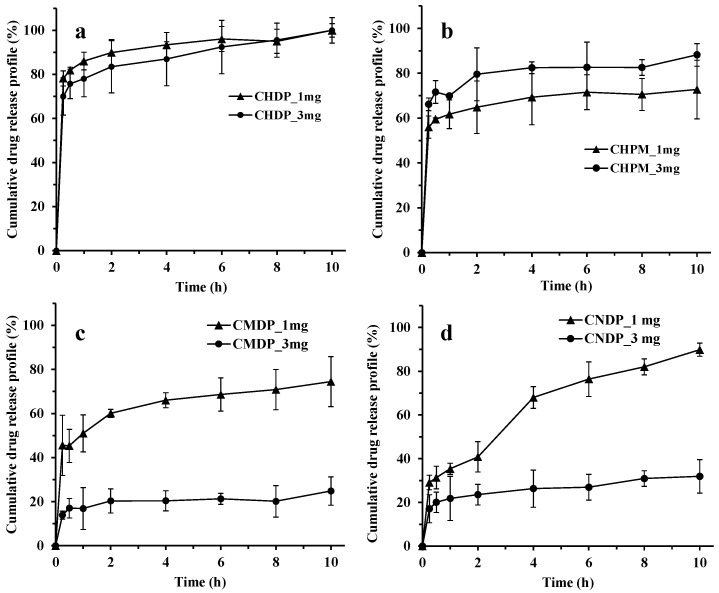
Drug release profiles from CHDP (**a**), CHPM (**b**), CMDP (**c**), and CNDP (**d**). Mean ± SD, n = 3. The masses represent the equivalent ciprofloxacin filled in capsules.

Drug release profiles from different amounts of the tested formulations are illustrated in Figure 4. According to the presented data, all formulations exhibited the initial burst drug release, yet the overall drug release was visibly decreased from CMDP and CNDP containing less soluble CIPB form compared to CHDP and CHPM containing more soluble CIPH form. This implies that the differences in solubility of CIPB and CIPH, especially under the applied non-sink test conditions, strongly influenced the drug release rate. The fastest drug release was observed from CHDP, reaching more than 80% drug release in 15 min, and about 90% drug release within 2 h (Figure 4a). CHPM also exhibited an initial burst drug release (60% in 15 min) (Figure 4b), which can be attributed to the high solubility of CIPH. Additionally, the presence of amorphous CIP forming in CHDP and CHPM particles (as confirmed by the PXRD results) contributes to the initial fast drug release from these formulations. After the initial burst phase, drug release from CHPM was retarded by the PLGA matrix, since PLGA is a hydrophobic polymer known to render extended drug release profile [29,30,31,32]. 

On the other hand, drug release rates from CMDP and CNDP were more gradual and largely depended on the mass of samples (Figure 4c,d). Namely, an increase in the mass of samples generally impeded drug release, so the cumulative amount of drug dissolved from 3 mg samples of these two formulations was rather low (less than 35% in 10 h). This may be explained by poor surface wetting of CIPB particles in the dissolution process. Moreover, the initial drug release rate from CMDP was somewhat faster compared to CNDP (i.e., 14–46% vs. 17–29% in 15 min, respectively), although CIP microparticles in CMDP were larger than CIP nanoparticles in CNDP. However, CMDP and CNDP were prepared using different drying techniques, which may explain the observed differences in drug release profiles. As observed in the SEM images, both CMDP and CNDP are aggregates of the primary particles. Nevertheless, in general, lyophilized particles are more porous compared to spray-dried particles [17]. Therefore, the initial faster drug release rate from CMDP might result from the high porosity of this sample. Still, the cumulative amount of drug release after 10 h is somewhat higher from CNDP samples (compared to CMDP), indicating that particle size governs drug dissolution following the initial burst phase. To better understand the implication of different drug release rates from the four formulations, their in vivo pharmacokinetic performance was investigated and reported in the last section.

### 3.4. In Vivo Lung Distribution

Regional lung deposition of the tested dry powder formulations was studied in a rat model, and the results are expressed as the percentage of Penn-Century aerosolizer delivered dose deposited in the trachea, bronchi, and alveoli (Table 2). The results show that the deposition patterns of CHDP and CHPM are similar, with a larger fraction of particles deposited in the alveolar region than in the bronchi.

In contrast, CMDP particles were deposited mainly in the bronchiolar region, and to a lesser extent in the alveoli. The deposition pattern of CNDP also differed from that of the other formulations. More than two-thirds of the particles in this formulation were deposited in the alveolar region, while bronchiolar deposition was approximately two times lower than for the other three formulations. These results are in agreement with the aerosol performance study conducted by the NGI. Namely, CNDP possesses the lowest MMAD and the highest FPF value, leading to the highest particle deposition in the lower airways.

### 3.5. In Vivo Pharmacokinetics

**Figure 5:**
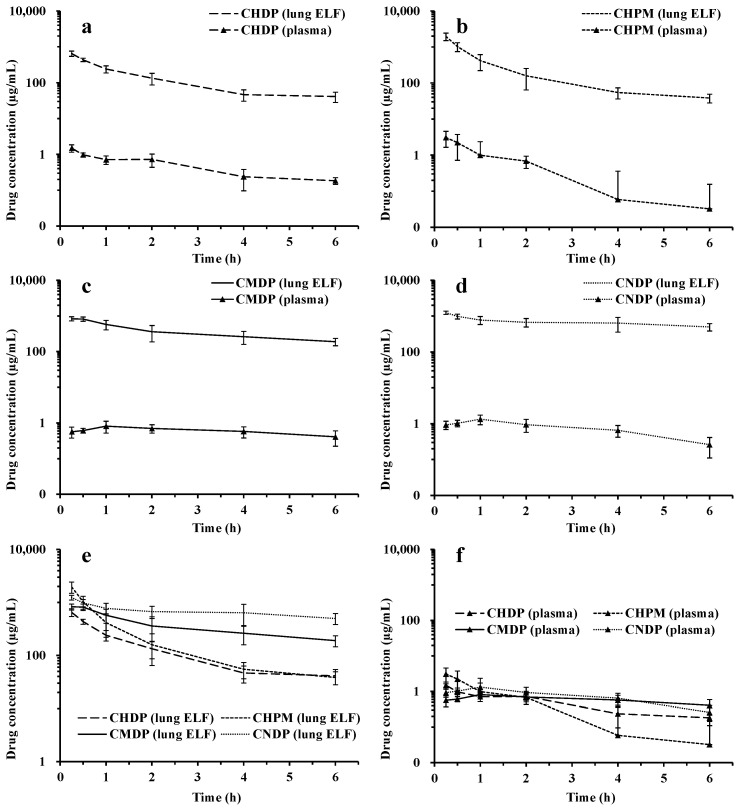
Lung ELF and plasma CIP concentration-time profiles of CHDP (**a**), CHPM (**b**), CMDP (**c**), CNDP (**d**), and merged graph (**e**,**f**) by intratracheal administration to rats. Mean ± SD, n = 4.

**Figure 6:**
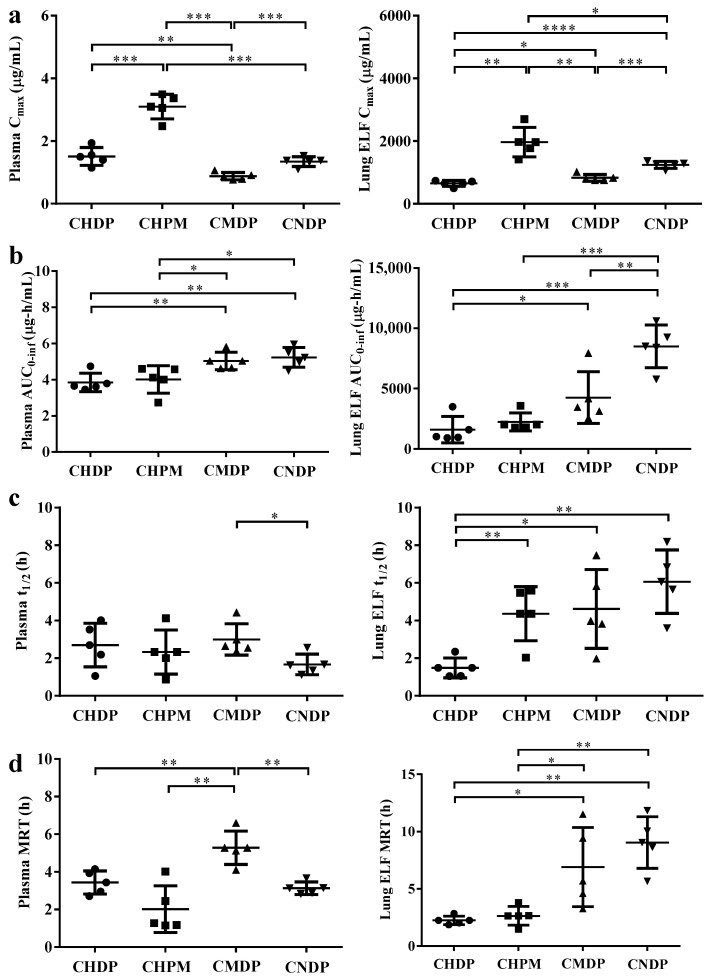
Pharmacokinetic parameters of CIP after intratracheal administration of four DPI formulations to rats: C_max_ (**a**), AUC_0-inf_ (**b**), t_1/2_ (**c**) and MRT (**d**). Statistical data by Student’s *t*-test with Welch’s correction: significant (* *p* < 0.05), very significant (** *p* < 0.01), highly significant (*** *p* < 0.001), extremely significant (**** *p* < 0.0001).

Pharmacokinetic studies were performed to evaluate the in vivo disposition of CIP following intratracheal administration of the four formulations to rats. The drug concentration-time profiles in plasma and ELF and the corresponding PK parameters are shown in Figure 5 and Figure 6, respectively. 

Figure 5 shows that peak drug concentrations (C_max_) in plasma following administration of CHDP and CHPM appeared at the first sampling time point (15 min), indicating rapid drug uptake into the systemic circulation. Besides the rapid initial drug release rate (Figure 4a,b), rapid drug absorption from these two formulations could be attributed to the high lipophilicity of CIP. CHPM exhibited lower drug plasma exposure (expressed as AUC_0-inf_) than CHDP, probably due to incomplete drug release from CHPM, where the PLGA matrix may retain a certain amount of the drug, resulting in lower systemic exposure. For CMDP and CNDP (Figure 5), the peak drug plasma concentration appeared at 1 h and thereafter the plasma concentrations of CIP declined. The C_max_ and AUC_0-inf_ of CMDP and CNDP are lower than that of post-intravenous injection and nebulization in our previous study [9]. It suggests that the systemic exposure of CIP has been largely suppressed when it was formulated into CIPB micro/nanocrystal solid forms compared to CIPH liquid formulations. In addition, the prolonged t_max_ for CMDP and CNDP compared with CHDP and CHPM most likely results from a slower drug release rate from micro- and nanocrystal formulations (Figure 4c,d).

CHPM exhibited higher lung exposure (expressed as AUC_0-inf_) than CHDP (Figure 6). In contrast to the rapid drug elimination from the systemic circulation, the pulmonary clearance of CIP following inhalation of the tested DPIs, especially CMDP and CNDP, was much slower. Moreover, CNDP exhibited the highest drug exposure in ELF (AUC_0-inf_), and the largest MRT between the tested formulations, which could be attributed to a large proportion of CNDP deposited in the alveolar region where mucociliary clearance (MCC) is not active [33]. 

To further describe the retention time of the drugs in the lungs, three indexes, i.e., the AUC_EPR_, AppELF, and PTI, were calculated and are listed in Table 3. The maximum AUC_EPR_ values for a nebulized solution or intravenous injection typically range from 1 to 50, as reported in previous studies [17,25] and also observed in our study [9]. The AUC_EPR_ values of a nebulized solution or intravenous injection range are 1.3 and 5.7, respectively. In contrast, the AUC_EPR_ of the tested formulations is rather high, beyond 200. In addition, the AppELF and PTI values for the four formulations are much higher than those of the nebulized solution. Such results imply that the lung retention time of the drug could be increased by formulating the drug into dry powder formulations. Although the lung deposition patterns of CHDP and CHPM are similar (Table 2), the AUC_EPR_ for CHPM is higher than that for CHDP. This could be attributed to the slower CIP release rate from CHPM compared to CHDP. The dissolved drug could be quickly absorbed across the air–blood barrier, shortening drug retention time in the lungs. The retention time of CNDP in the lungs is the longest among the four DPIs, and this could be partly attributed to the slow drug release rate from this formulation (Figure 4d).

In addition, the data in Table 2 show that the majority of CNDP was deposited in the lower part of the lung, whereas the other formulations were mostly deposited in the middle airways. Therefore, there might be less mucociliary clearance of CNDP in comparison to the other formulations, resulting in the prolonged residence of CNDP in the lungs. Overall, the results suggest that lung exposure of highly permeable drugs could be increased and prolonged using sustained-release formulation strategies regardless of the polymer-based system (CHPM) and the solubility-limited particles (CNDP and CMDP).

## 4. Conclusions

In this study, an antibiotic model drug (ciprofloxacin) was formulated into four DPIs via different formulation strategies. Compared to the immediate-release formulation (CHDP), PLGA-based sustained-release microparticles (CHPM) and dissolution rate-controlled formulations (CMDP and CNDP) could improve the lung exposure of the drug via post-pulmonary administration. In addition, the retention of the drug in the lung (ELF) can be prolonged via a sustained-released formulation strategy and by lowering the dissolution rate. This study also demonstrates the feasibility of formulating a highly permeable antibiotic into inhalable sustained-release dry powders with improved lung bioavailability. However, the antibacterial efficiency of these sustained-release formulations should be assessed in future studies on lung infection models.

## Figures and Tables

**Table 1:** Aerosol properties of CHDP, CHPM, CMDP, and CNDP. Mean ± SD, n = 3.

Formulation	EF (%)	MMAD (µm)	GSD	FPF (%)
CHDP	97.15 ± 0.17	4.42 ± 0.32	1.80 ± 0.01	46.98 ± 6.17
CHPM	96.07 ± 0.42	4.53 ± 0.01	1.84 ± 0.20	37.39 ± 2.55 *
CMDP	96.68 ± 0.19	3.83 ± 0.47	2.78 ± 0.13 *	30.95 ± 5.01 **
CNDP	98.18 ± 0.17	2.24 ± 0.01 **	1.65 ± 0.03	71.81 ± 2.11 ***

Note: statistical analysis by Student’s *t*-test vs. CHDP, *p* < 0.05 significant (*), *p* < 0.01 very significant (**), *p* < 0.001 highly significant (***).

**Table 2:** In vivo regional lung deposition profiles post-intratracheal dosing to rats. Mean ± SD, n = 3.

Region/Deposition	CHDP (%)	CHPM (%)	CMDP (%)	CNDP (%)
Trachea	7.75 ± 2.96	4.76 ± 0.78	9.41 ± 8.87	12.03 ± 1.63
Bronchi	41.34 ± 14.11	43.60 ± 8.63	49.76 ± 18.60	20.91 ± 2.72
Alveoli	50.91 ± 13.15	51.64 ± 7.2	40.83 ± 12.16	67.07 ± 9.22

**Table 3:** Calculated parameters describing in vivo distribution (AUC_EPR_), lung bioavailability (AppELF), and pulmonary target index (PTI) for CIP after dosing the tested formulations in rats.

Formulation	AUC_EPR_	AppELF	PTI
i.v. injection **^#^**	1.30	NA	NA
i.t. solution **^#^**	5.71	4.76	4.39
i.t. CHDP	278.21	153.30	213.65
i.t. CHPM	427.03	285.05	327.94
i.t. CMDP	503.22	401.18	386.45
i.t. CNDP	881.81	737.16	677.19

^#^ Cited from [9]; NA, not available; i.v. intravenous drug delivery; i.t. intratracheal drug delivery.

## Data Availability

The data that support the findings of this study are available from the authors upon reasonable request.
